# Machiavellianism and Adult Attachment in General Interpersonal Relationships and Close Relationships

**DOI:** 10.5964/ejop.v11i1.801

**Published:** 2015-02-27

**Authors:** Tamás Ináncsi, András Láng, Tamás Bereczkei

**Affiliations:** a Department of General and Evolutionary Psychology, University of Pécs, Pécs, Hungary; b Department of Personality, Development and Clinical Psychology, University of Pécs, Pécs, Hungary; Academy of Special Education, Warsaw, Poland

**Keywords:** Machiavellianism, dismissing-avoidant attachment style, attachment-related avoidance and anxiety, general interpersonal relationships and close relationships

## Abstract

Up to the present, the relationship between Machiavellianism and adult attachment has remained a question to be answered in the psychological literature. That is why this study focused on the relationship between Machiavellianism and attachment towards significant others in general interpersonal relationships and in intimate-close relationships. Two attachment tests (Relationship Questionnaire and long-form of Experiences in Close Relationship) and the Mach-IV test were conducted on a sample consisting of 185 subjects. Results have revealed that Machiavellian subjects show a dismissing-avoidant attachment style in their general interpersonal relationships, while avoidance is further accompanied by some characteristics of attachment anxiety in their intimate-close relationships. Our findings further refine the relationship between Machiavellianism and dismissing-avoidant attachment. Machiavellian individuals not only have a negative representation of significant others, but they also tend to seek symbiotic closeness in order to exploit their partners. This ambitendency in distance regulation might be particularly important in understanding the vulnerability of Machiavellian individuals.

In recent years, rapid growth occurred in the number of studies addressing the Dark Triad and, within that, Machiavellianism (Jonason & Krause, 2013; Jones & Weiser, 2014). Machiavellianism has already been investigated in several contexts. Still, little is known about the way Machiavellians are attached to their partners in romantic relationships. While Machiavellians are especially skilled and successful in deceiving others in everyday interpersonal situations such as those involving competition and strategy (Czibor & Bereczkei, 2010), the case is completely different with social contexts aimed at trust, commitment and cooperation (Pilch, 2008; Pilch, 2012). This study attempts to fill in the mentioned gap in the literature by exploring Machiavellian individuals’ adult attachment patterns shown in general interpersonal relationships and intimate-close relationships. In this way, Machiavellians’ views on interpersonal relationships are approached at both a more general and a closer level.

## Machiavellianism

The concept of Machiavellianism originates from the name Niccolo Machiavelli (1469-1527) whose concept became over the course of time inseparably associated with the conception of a “*dark personality*” and “*the dark side of love*” (Ali & Chamorro-Premuzic, 2010; Hatfield, 1984; Shaver & Hazan, 1988). Machiavellian individuals may be described by such characteristics as deceitful, dissimulative, false-hearted, manipulative, self-seeking, exploitative, utilitarian, cynical, indifferent, emotionally cold, unscrupulous, hypercompetitive and egocentric.

Christie and Geis (1970) suggest that the primary characteristic differentiating between less and more Machiavellian individuals (referred to as low and high Machs) is the measure of emotions invested in interpersonal relationships. The *emotionally detached interpersonal orientation* is an essential factor of Machiavellianism to the extent that Christie and Geis (1970) described high Machs by the term “*cool syndrome*” as opposed to low Machs described by the term “*soft touch*”. This differentiation is supported by the results of Wastell and Booth (2003) suggesting that Machiavellians are characterised by alexithymia, that is, their inner experiences are poor, they live in an emotionally vacant world and have no connection to their own emotions. As a result of being unaware of their own emotional experiences, they are unable to empathetically attune to others (Andrew, Cooke, & Muncer, 2008; Barnett & Thompson, 1985; Jakobwitz & Egan, 2006; Paál & Bereczkei, 2007) and to provide social support. Their difficulties in social emotional cognition are illustrated by a study of Austin and colleagues (2007) who have found that Machiavellians show a low level of emotional intelligence.

## Etiology of Dismissing-Avoidant Attachment Style and Machiavellianism

Dismissing-avoidant individuals’ parents are emotionally inexpressive and unavailable in a psychological sense. They do not provide a feeling of security, they cannot soothe their child when she or he in distress, and they avoid close physical contact (Cassidy & Kobak, 1988; Fraley, Davis, & Shaver, 1998). These parents disapprove and undervalue the experience and expression of emotions, and consider attachment behaviour to be childish and a sign of weakness. They moreover expect autonomy and achievements from their children rather than establishing intimate relationships with others (Bartholomew, 1990). As a consequence, avoidant individuals learn over time that the experience of emotions is harmful (Montebarocci, Codispoti, Baldaro, & Rossi, 2004), intimate interactions with others are painful and they can only expect refusal. In order to avoid the pain of loss and disappointment, they strive to prevent psychological closeness, intimacy, and considerable investments in close relationships and to keep the attachment system deactivated (Mikulincer & Shaver, 2003; Shaver & Mikulincer, 2002). As they pay little attention to emotional experiences (Fraley & Brumbaugh, 2007), they are not influenced by them and this condition may be described as alexithymia (Montebarocci et al., 2004; Troisi, D’Argenio, Peracchio, & Petti, 2001).

Several studies suggest that the etiology of Machiavellianism, similarly to the development of a dismissing-avoidant pattern, partly originates from childhood experiences obtained in relationships with unexpressive, less understanding, highly punitive or restrictive caregivers (Christie & Geis, 1970; Guterman, 1970; Jonason, Lyons, & Bethell, 2014; Kraut & Lewis, 1975; Ojha, 2007; Touhey, 1973). Thus, apparently, it may also be established from a developmental aspect, following the observation of Wastell and Booth (2003), that the Machiavellian attitude is not based on voluntary choice but is a constrained developmental path depending on childhood experiences. Other studies (e.g. Láng & Birkás, 2014) highlight chaotic functioning in the family of origin of Machiavellian individuals.

## Internal Mental Working Model

### Working Models of Dismissing-Avoidant Individuals

Attachment and romantic relationships are fundamentally based on mental models or emotional schemata of the representational world (Sandler & Rosenblatt, 1962). These cognitive concepts refer to the process in which one forms a model or image of the self, a model of others as well as a model of the rules on which interpersonal relationships are based (Bowlby, 1973, 1980, 1982).

Dismissing-avoidant individuals’ model of self is characterised by a positive self-image (Bartholomew & Horowitz, 1991). Bartholomew (1990; p. 164) suggests that “A way of maintaining a positive self-image in the face of rejection by attachment figures is to distance oneself and develop a model of the self as fully adequate and hence invulnerable to negative feelings …”. Since their self-esteem is to a great extent internalised, they do not need external reinforcement and, by establishing rigid self-boundaries, they show self-confidence and independence even when facing permanent losses (Fraley & Shaver, 1999). Bowlby (1973) and other authors termed such a disposition as “*compulsive self-reliance*” or an “*excessive need for self-esteem*”. They regularly describe themselves in positive terms and restrict cognitive access to negative self-traits (Mikulincer, 1995) and consider themselves rather than others as a basis of security (Fraley & Davis, 1997).

Dismissing-avoidant individuals tend to maintain models of others in which they are represented as malevolent, unreliable and hostile. As dismissing-avoidant individuals’ trust in others is unstable (Collins & Read, 1990; Feeney & Noller, 1990; Simpson, 1990), they hold negative expectations on others’ availability and hold the belief that one cannot rely on significant others in times of crisis. They are distrustful towards others’ intentions, therefore they are not willing to either provide or accept support and care (Collins & Feeney, 2000; Simpson, Rholes, & Nelligan, 1992).

According to dismissing-avoidant individuals’ models of close relationships, they hold that the real one does not actually exist, only in novels and films. Romantic relationships do not last long and happiness does not require maintaining close relationships (Feeney, 1999; Levy & Davis, 1988; Shaver & Hazan, 1988).

Researchers have found that dismissing-avoidant attachment style is negatively related to all three components of the love triangle of Sternberg (1986, 1988) (Levy & Davis, 1988) as well as to interpersonal closeness (Brennan et al., 1998; Collins & Read, 1990). It is also negatively related to interpersonal dependence, trust (Simpson, 1990), and self-disclosure (Mikulincer & Nachshon, 1991). Dismissing-avoidant individuals are characterised by a preference for casual sex (Brennan et al., 1998; Feeney, Noller, & Patty, 1993) and a sociosexual orientation lacking intimacy (Brennan & Shaver, 1995). They do not particularly adhere to their partners and especially dismissing-avoidant men experience less anxiety following the break-up of a relationship (Simpson, 1990; Sprecher, Felmlee, Metts, Fehr, & Vanni, 1998).

### Working Models of Machiavellians

High Machs’ self-models are characterised by rigid self-boundaries, compulsive self-reliance and stable self-esteem. They evaluate their autonomy and independence highly, whereby their need for psychological closeness is minimalised. They only show their abilities and positive attributes to the social environment while concealing their feelings and personal deficiencies based on the belief that disclosing emotions and vulnerability is a sign of weakness that would merely be an invitation to others to exploit them (Sherry, Hewitt, Besser, Flett, & Klein, 2006).

One major characteristic of a model of others based on a Machiavellian attitude is a negative, cynical view on human nature (McHoskey, Worzel, & Szyarto, 1998) and generalised distrust against others. Machiavellians conceive of others as unpredictable and unreliable. Led by a paranoid suspicion, Machiavellian individuals perceive others as pursuing ill intentions, as controlling, demanding, hostile and threatening. They are unable to find values that could be respected in their parents, partners or others.

With respect to relational models, high Machs hold that intimacy is risky because trust is always subject to betrayal and self-disclosure incurs especial vulnerability. Therefore, they avoid deep intimacy and are reluctant to share their ideas and feelings with others. Due to their utilitarian and instrumentalist attitude, they enter into short-term relationships at a low investment rate (Holtzman, 2013; Jonason, Li, Webster, & Schmitt, 2009) and they are not interested in establishing committed, emotionally deep relationships. They maintain an impersonal relation to their partners, only treating them as exploitable objects. Their sexual behaviour is characterised by promiscuous, *unrestricted sociosexual orientation* (McHoskey, 2001a). Thus, high Machs focus their resources on personal goals rather than on people and overemphasise the extrinsic motivation of material success as opposed to such intrinsic motivations as community and family (McHoskey, 1999). Ali and Chamorro-Premuzic (2010) have found that Machiavellianism is negatively related to the intimate relationship dimensions of Sternberg (1986, 1988), that is, intimacy and commitment.

## Machiavellianism, Dismissing-Avoidant Attachment Style and Psychological Vulnerability

If they are to be represented on a bipolar dimensional scale whose poles are seeking for closeness (attachment) and avoiding closeness (autonomy), both dismissing-avoidant and Machiavellian individuals should be placed on the emotionally cold pole of the scale (Bekker, Bachrach, & Croon, 2007; Jones & Paulhus, 2011; Zimberoff & Hartman, 2002). Despite being characterised by low anxiety besides high avoidance, dismissing-avoidant individuals’ invulnerability is questionable. Although dismissing-avoidant individuals mostly adapt well to everyday life and report psychological balance, this is much more due to their powerful defense mechanisms than to their actual psychological well-being. This is also reflected in the results of studies (Mikulincer, Dolev, & Shaver, 2004) which have revealed that avoidant subjects’ defense strategies prove to be vulnerable and may are likely to collapse in situations involving intense and permanent distress (Dozier & Kobak, 1992). Studies have demonstrated that dismissing-avoidant individuals may tend to experience negative mood states such as depression or loneliness (Wei, Russel, Mallinckrodt, & Vogel 2007) and, furthermore, hostile emotions (Kobak & Sceery, 1988) neuroticism (Conradi, Gerlsma, van Duijn, & de Jonge, 2006), less frequent positive emotions and more frequent negative emotions (Mikulincer & Nachshon, 1991; Simpson, 1990) and that they may also tend to more quickly recall words with negative contents (Baldwin, Fehr, Keedian, Seidel, & Thomson, 1993), apply less constructive and less flexible emotion regulation strategies (Block & Block, 1980; Kobak & Sceery, 1988) and maladaptive coping strategies such as alcohol consumption (Brennan & Shaver, 1995; Brennan, Shaver, & Tobey, 1991).

Evidence for a similar vulnerability concerning Machiavellians has also been reported in the literature. High Machs have dysfuncitional personalities; they are characterised by unbalanced emotional functioning, hostile and negative attitudes, and depressive symptoms (Jakobwitz & Egan, 2006; McHoskey, 2001b; McHoskey et al., 1998; Paulhus & Williams, 2002).

The above description may raise the following question: how much overlapping or perhaps identical constructs are dismissing-avoidant attachment and Machiavellianism? In our opinion, Machiavellianism is only one of the possible ways of coping with a rejecting, abusing or neglecting social environment. While some of the dismissing-avoidant individuals respond to childhood adversities with withdrawal from the social world, Machiavellian individuals try to cope with expected refusals and hurts by means of their controlling and deceptive interpersonal tactics.

## Hypotheses

Given that that both dismissing-avoidant individuals and Machiavellians form such cognitive models of themselves, their partners and close relationships which emphasise self-confidence, distrust of others and a defensive interpersonal style against psychological closeness, we formulate the following hypotheses:

1. In general interpersonal relationships, high Machs are attached in a dismissing-avoidant style whereas low Machs are securely attached.

2. In intimate-close relationships, Machiavellianism is positively related to avoidant attachment. Since the relevant literature is characterised by uncertainty regarding anxiety, no specific hypotheses were formed in this study concerning the relationship between Machiavellianism and attachment anxiety.

## Method

### Sample

Second-, third- and fourth-year law students of the University of Miskolc, Hungary and the University of Pécs, Hungary participated in the study. The overall sample consisted of 210 subjects. 15 subjects were excluded from the overall sample due to incomplete filling of the questionnaires and 12 additional subjects were excluded because they reported that they had never had an intimate partner. Exclusion of the latter was necessary because the *Experiences in Close Relationships Questionnaire (ECR)* is only capable of measuring attachment of those who have already had a romantic relationship. Without relevant experience, subjects are not able to give valid responses. In this way, the actual sample consisted of 183 subjects (53 male; 130 female). Subjects’ age varied between 19 and 27 years. (*M* = 21.63; *SD* = 1.57).

### Measures

Hypothesis testing was based on self-report measures. The two attachment questionnaires presented below were selected primarily because they enabled the assessment of attachment and relationship attitudes towards both less close general relationships and romantic relationships. Furthermore, the two questionnaires also differ in their underlying methodologies: one is based on attachment categories while the other is based on attachment dimensions. The authors of this study suggest that the joint application of the questionnaires enables a more precise assessment.

#### Relationship Questionnaire – RQ (Bartholomew & Horowitz, 1991) 

Based on the *Adult Attachment Questionnaire* developed by Hazan and Shaver (1987), Bartholomew and Horowitz (1991) developed a forced-choice instrument comprising separate items which measures adults’ general willingness to be attached. The scale describes four prototypical attachment patterns such as secure, dismissing-avoidant, anxious-preoccupied and fearful-avoidant, defining the major characteristics of the four attachment styles. Subjects were first asked to evaluate each category on a 7-point Likert scale marking the value which best reflected how they acted and felt in general interpersonal relationships. Then they were asked to select the prototype which most characterised them according to the previously presented descriptions.

#### Experiences in Close Relationship – ECR (Brennan, Clark, & Shaver, 1998)

Currently, the most widely accepted attachment model is the typology comprising four attachment styles (Bartholomew, 1990; Bartholomew & Horowitz, 1991) based on two underlying latent dimensions. One dimension is the self-model, also referred to as anxiety, that represents a particular view on the valuableness of the self. The other dimension is the model of the other, also referred to as avoidance, that includes general expectations on others’ availability, responsiveness and reliability (Griffin & Bartholomew, 1994). Attachment avoidance describes the intensity of attachment such as the feeling of discomfort related to closeness or the avoidance of intimacy. Attachment anxiety describes the quality of attachment such as being worried about the durability of relationships or being anxious about rejection or abandonment (Brennan et al., 1998). The scale has been designed to assess the general pattern of adult romantic attachment, i.e. the way subjects experience their close relationships in general and not the feelings they experience in their current relationship. Brennan and colleagues developed the 142-item ECR combining adult attachment scales available in 1997. The first 36 items of the scale define a factor structure of two continuous attachment dimensions, one of which measures *attachment avoidance* and the other measures *attachment anxiety*. The extended version of the scale assesses 12 additional attachment-related dimensions besides the two principal dimensions. These additional scales enable a more specific assessment of avoidance and anxiety (each measured by six dimensions) and a more accurate indication of individual differences in adult romantic attachment. Subjects indicated their responses on a 7-point Likert scale ranging from do not agree at all (1) to agree absolutely (7). The reliability of ECR dimensions ranged from .74 to .92 (Cronbach’s alphas).

#### Mach IV (Christie & Geis, 1970)

The 20-item Mach scale was designed by social psychologists of the Columbia University to assess the propensity for fraud and interpersonal manipulation as well as cynical and unmoral attitudes and beliefs. Ten items each reflect the acceptance and ten items the avoidance of a Machiavellian attitude. The Mach score is the sum of the ratings given to the 20 items plus 20. Each item of the scale is followed by a 7-point Likert scale ranging from do not agree at all (1) to agree absolutely (7). The minimum value of Machiavellianism is 40, the maximum is 160. Higher scores on the scale indicate higher levels of Machiavellianism. Subjects were divided into two groups based on the median (98) of Mach-IV in our sample: (1) low Machs (70 – 97); (2) high Machs (98 – 135). Mach IV proved to be a reliable instrument in our study (Cronbach’s alpha = .73).

### Procedure

The survey was conducted in university classrooms where subjects filled in the questionnaires alone after receiving instructions. Data were collected in groups of subjects. Participation was anonymous to prevent subsequent personal identification of subjects. In order to ensure secrecy and prevent dissimulation, subjects were informed before the survey that they should individually place their filled out questionnaires in a large envelope circulated in the classroom at the end of the session. Subjects participated in the study voluntarily, without receiving course credits or financial compensation.

## Results

### Relationship Between Machiavellianism and Attachment Style in General Interpersonal Relationships

At the first stage of data analysis, a Chi-square test was conducted to compare frequencies of different general attachment styles assessed by the *Relationship Questionnaire* (RQ) in the low Mach and high Mach groups. The result of the Chi-square test showed that the distribution of attachment styles was not independent of Machiavellianism (χ^2^(3) = 9.612; *p* < .05). Distributions reported in Table 1 show that the significant difference from random distributions resulted from the significantly higher frequency of securely attached subjects among low Machs as well as from the significantly higher frequency of dismissing-avoidant subjects among high Machs.

**Table 1:** Distribution of Subjects With Different Attachment Styles in the Low Mach and High Mach Groups

Group	Attachment style			
	Secure	Dismissing	Preoccupied	Fearful
Low Mach	*n* = 39 (66%)	*n* = 16 (37%)	*n* = 14 (45%)	*n* = 26 (53%)
High Mach	*n* = 20 (34%)	*n* = 28 (63%)	*n* = 17 (55%)	*n* = 23 (47%)
Total	*n* = 59 (100%)	*n* = 44 (100%)	*n* = 31 (100%)	*n* = 49 (100%)

At the second stage, the low and high Mach groups were compared in an analysis of the variance in their ratings of each attachment style they were presented with in descriptions. Results are shown in Table 2. Data show that low and high Machs only differed in the evaluation of one attachment style, namely, dismissing-avoidant attachment (Cohen’s *d* = .49). High Machs evaluated the description of dismissing-avoidant attachment as characterising them to a significantly higher degree than their low Mach counterparts.

**Table 2:** Comparisons Between the Low Mach and High Mach Groups in the Dimensional Evaluations of the Four Attachment Styles; Results of the Analyses of Variance

Attachment Style	Low Mach		High Mach		*F*	*p*
	*M*	*SD*	*M*	*SD*		
Secure	4.12	1.60	3.98	1.57	0.347	.557
Dismissing	3.06	1.75	3.99	2.00	11.029	.001
Preoccupied	3.33	1.67	3.52	1.71	0.581	.447
Fearful	3.53	1.93	3.84	2.01	1.167	.282

*Note. df* = 1,182 in each case.

### Relationship Between Machiavellianism and Attachment Dimensions in Close Interpersonal Relationships

At the third stage of data analysis, the relationship between Machiavellianism and adult romantic attachment dimensions – measured by *Experiences in Close Relationships Questionnaire (ECR)* – was tested with Pearson’s correlations in order to better fit the nature of our data (i.e., interval scale; Table 3). According to the results of Pearson’s correlations individuals with more pronounced Machiavellian attitudes showed more avoidant attachment in their romantic relationships. This relationship was also reflected in all six additional avoidant attachment dimensions. More Machiavellian individuals conceived of their partner as a worse attachment figure, trusted their partner less, and reported themselves to be more self-reliant. They also reported higher incidence of discomfort both with closeness and dependence, and claimed to hang on to their independence tough-mindedly. Although the principal dimension of attachment anxiety was not significantly correlated with Machiavellianism, four out of six additional anxious attachment dimensions showed a significant relationship with Mach-IV scores. Thus, more Machiavellian individuals showed less separation anxiety, more attachment-related anger at partner, more uncertainty about their feelings towards their partners. They also uttered a more pronounced desire to merge with their partners.

**Table 3:** Pearson’s correlations between Machiavellianism and the two higher order and 12 lower order dimensions of ECR

Dimension	1	2	3	4	5	6	7	8	9	10	11	12	13	14
ECR higher order dimensions														
1. Avoidance	-													
2. Anxiety	.15*	-												
ECR lower order dimensions														
3. Partner is a Good Attachment Figure	-.58**	-.24**	-											
4. Self-Reliance	.62**	-.04	-.41**	-										
5. Discomfort with Closeness	.90**	.17*	-.57**	.57**	-									
6. Discomfort with Dependence	.77**	.08	-.48**	.65**	.68**	-								
7. Trust in Partners	-.54**	-.37**	.45**	-.46**	-.55**	-.57**	-							
8. Tough-Minded Independence	.62**	-.17*	-.46**	.66**	.62**	.62**	-.37**	-						
9. Separation Anxiety	-.51**	.37**	.46**	-.49**	-.46**	-.37**	.15*	-.56**	-					
10. Attachment-Related Anger at Partners	.34**	.58**	-.37**	.19**	.41**	.24**	-.46**	.15*	.11	-				
11. Uncertainty About Feelings for Partners	.66**	.28**	-.57**	.43**	.72**	.44**	-.45**	.50**	-.37**	.57**	-			
12. Lovability / Relational Self-Esteem	-.47**	-.45**	.48**	-.18*	-.48**	-.32**	.44**	-.13	.06	-.39**	-.40**	-		
13. Desire to Merge with Partners	.41**	.81**	-.48**	.18*	.44**	.32**	-.49**	.15*	.10	.61**	.45**	-.53**	-	
14. Fear of Abandonment	.38**	.76**	-.42**	.21**	.37**	.27**	-.47**	.07	.13	.53**	.45**	-.63**	.74**	-
**Machiavellianism**	.33**	.12	-.33**	.28**	.28**	.34**	-.53**	.31**	-.23**	.23**	.19**	-.11	.21**	.10

*p < .05. **p < .01.

## Discussion

This study was aimed at exploring relationship between Machiavellianism and attachment-related attitudes in general interpersonal relationships and intimate-close relationships. Concerning attachment measured in relation to general interpersonal relationships, results showed that dismissing-avoidant attachment style and Machiavellianism were closely related constructs. Specifically, the distribution of different attachment styles between the two Mach groups showed that most high Machs were found among dismissing-avoidant subjects while most low Machs were securely attached. Furthermore, high Machs found dismissing-avoidant attachment to be characteristic to them to a significantly higher degree than did their low Mach peers. These results confirm Hypothesis 1.

Results obtained from the dimensional measures of ECR showed a more sophisticated picture of the relation between Machiavellianism and attachment in an intimate-close relationship. According to the results, Machiavellianism was positively correlated with attachment avoidance, but was uncorrelated with attachment anxiety. The robust finding between Machiavellianism and attachment avoidance was further supported by significant correlations between Machiavellianism and each additional attachment avoidance dimension. These results support Hypothesis 2. Nevertheless, additional attachment anxiety scales showed that angry feelings toward the partner, uncertainty about feelings towards partner, desire for merging, and relative lack of separation anxiety are also characteristic of Machiavellian individuals (Aim 1).

Our results partially replicate those describing Machiavellian individuals as having an emotionally detached interpersonal orientation (Christie & Geis, 1970; McHoskey, 1999; Wastell & Booth, 2003). Reflected in dismissing-avoidant attachment, Machiavellian individuals have a positive model of self and a negative model of others. Others are viewed as malevolent and as people who cannot be trusted (Christie & Geis, 1970). So, at the level of internal working models, dismissing-avoidant attachment and Machiavellianism seem to overlap. At the same time, Machiavellianism’s relation with attachment anxiety highlights that means of coping with interpersonal situations – especially in intimate-close relationships – differ for Machiavellian and dismissing-avoidant individuals. Put in a more precise way, the Machiavellian strategy can be one of several strategies that help individuals to cope with anxiety resulting from rejection by caregivers or romantic partners. Insensitive of separation itself, Machiavellian individuals want to merge with their partners in order to control them. Their anger at their partner also results from the uncontrollable actions of the partner. Uncertainty of their feelings towards the partner might be a result of their alexithymia (Wastell & Booth, 2003).

Apparently, Machiavellians’ interpersonal attitude is based on a symbiotic-hostile merging with the partner rather than simple interpersonal distance. Machiavellians’ relationships are symbiotic in the sense that they consider their partners to merely be exploitable objects whose existence exclusively depends on their ability to satisfy Machiavellians’ self-related needs (Paál & Bereczkei, 2007). Led by utilitarianism, they want to get but not give in romantic relationships. At the same time, such relationships are also hostile since Machiavellians manipulate their partners against their partners’ will, and this exploitative behaviour restricts partners’ personal prospects and growth. Machiavellians practically live at their partners’ expense. When partners no longer provide any benefits or actively deny further exploitation, they may elicit devastating anger from Machiavellians who then abandon the relationship in order to prevent further frustration. Machiavellians’ greatest fears result from losing control over their partners. This may occur frequently as a result of abandonment because people often choose to escape from a relationship established with an untruthful, manipulative and exploitative person (Anderson, 1968; Buss, 1989; Fletcher, Simpson, Thomas, & Giles, 1999). Machiavellian individuals unrestricted sociosexual orientation also increases the likelihood of being abandoned (McHoskey, 2001a). Machiavellians respond with intense anger to any suspicion of abandonment or being let down. This is done in a relatively immature manner by derogating the partner and attachment – all parts of an avoidant attachment strategy.

The symbiotic-hostile nature of Machiavellians’ intimate relationships as outlined above is seemingly contradicted by the observation that Machiavellians are irritated by their partners’ excessive need for closeness. This contradiction may also be due to their concealed vulnerability on the one hand, while, on the other hand, it may also indicate a form of need for control emerging in their relationships. Namely, Machiavellian individuals want to have exclusive control of closeness and distance in their relationships. By doing so, they transgress an essential rule of relationships, namely, reciprocity. Thus, it is not surprising that their relationships are short-lived and mostly provide little contentment (Ali & Chamorro-Premuzic, 2010).

To summarise the novel findings of our research, the above described pattern of additional attachment anxiety dimensions – an urgent need for merging, feelings of anger and frustration towards the partner with a simultaneous lack of separation anxiety – suggest that high Machs almost exclusively consider closeness as an opportunity for exploitation and control. Thus, from the perspective of object relations theory, Machiavellians’ close relationships are actually symbiotic in nature but such relationships provide a negative, destructive form of symbiosis (Hamilton, 1988). Furthermore, need for interpersonal distance resulting from the negative representation of others and the need for physical closeness that is necessary for control and exploitation are present in Machiavellian individuals at the same time. In our opinion, this makes Machiavellian individuals extremely vulnerable because the above mentioned contradiction between distance and closeness results in heightened levels of anxiety that can only be solved by the means of primitive defence mechanisms.

### Limitations and Prospects for Future Research

The study is not free from limitations. Firstly, the sample exclusively comprised university students, thus the results obtained cannot be generalised to a wider population. Secondly, the unbalanced distribution of genders in the sample might distort results. The overrepresentation of women might contribute to Machiavellians’ high score in the anxiety dimension of ECR (Del Giudice, 2011). Thirdly and finally, the study was conducted using a questionnaire methodology and was based on a cross-sectional arrangement. Consequently, the causal relationships established often remain hypothetical although the relationships revealed between the studied variables were obtained by means of accurate statistical analyses. A repetition of the study using projective tests or including real-life couples in the sample under laboratory conditions might bring especially intriguing results. A longitudinal study would enable us to observe how the degree of Machiavellianism measured before the establishment of an intimate partner relationship influences the course of romantic relationships from adolescence.

Subsequent studies should importantly apply more refined measures to investigate the vulnerability of Machiavellian individuals. Among others, it should be clarified whether Machiavellians – while staying physically close to others – are afraid of or indifferent to emotional closeness.
